# New endemic foci of tick-borne encephalitis (TBE) identified in districts where testing for TBE was not available before 2009 in Poland

**DOI:** 10.1186/1756-3305-6-180

**Published:** 2013-06-18

**Authors:** Pawel Stefanoff, Anna Zielicka-Hardy, Maria Hlebowicz, Ryszard Konior, Dariusz Lipowski, Leszek Szenborn, Joanna Siennicka, Hana Orlikova, TBE enhanced surveillance working group

**Affiliations:** 1 National Institute of Public Health-National Institute of Hygiene, Chocimska 24, 00-791 Warsaw, Poland; 2 European Programme for Intervention Epidemiology Training (EPIET), European Centre for Disease Prevention and Control (ECDC), Tomtebodavägen 11a, 171 83 Stockholm, Sweden; 3 Gdańsk Medical University, Marii Skłodowskiej-Curie 3A, 80-210 Gdańsk, Poland; 4 John Paul 2nd Hospital in Kraków, Prądnicka 80, 31-202 Kraków, Poland; 5 Medical University of Warsaw, Żwirki i Wigury 61, 02-091 Warsaw, Poland; 6 Wrocław Medical University, Wybrzeże Ludwika Pasteura 1, 50-367, Wrocław, Poland; 7 National Institute of Public Health, Šrobárova 48, 100 42 Prague, Czech Republic

**Keywords:** Tick-borne encephalitis, Surveillance, Endemic foci, Poland

## Abstract

**Background:**

Tick-borne encephalitis (TBE) is found in limited endemic foci in Poland. Lack of diagnosis limits disease detection in non-endemic provinces.

**Methods:**

In 2009, we enhanced TBE surveillance to confirm the location of endemic foci and inform vaccination policy. In 105 hospitals located in 11/16 provinces, we identified suspected TBE cases through admission ICD-10 codes indicating aseptic meningo-encephalitis or from specimens tested for TBE. The National Reference Laboratory confirmed cases at no cost, by testing serum and/or cerebrospinal fluid using ELISA method. We calculated TBE reported rates as the number of confirmed TBE cases per 100,000 inhabitants. Adjusting to neighbouring districts, we classified districts as non-endemic (<0.1 cases per 100,000 inhabitants), low endemic (> = 0.1 to <1), moderately endemic (> = 1 to <5) and highly endemic (> = 5). We compared surveillance data obtained in 2009 with 2004–2008 baseline data.

**Results:**

Among 166,099 admissions, we identified 1,585 suspected TBE cases of which 256 were confirmed. Physicians reported more suspected cases among patients <40 years old (12 cases per 1,000 admissions) than among older patients (8 cases per 1,000 admissions). However, patients <40 years of age were confirmed less frequently (16%), than older patients (35%). Physicians reported more suspected cases in districts classed as endemic during 2004–2008 (12 cases per 1,000 admissions, 77% tested for TBE) than in districts classed as non-endemic (7 cases per 1,000 admissions, 59% tested). Of the 38 newly identified endemic districts, 31 were adjacent to 2004–2008 endemic districts and 7 were isolated.

**Conclusions:**

Enhanced surveillance detected 38 new endemic districts to be considered for TBE vaccination. However, lack of consistent testing in districts believed to be TBE-free remained an obstacle for mapping TBE risk. Although the disease affects mostly older adults and the elderly, more attention is given to the diagnosis of TBE in young patients. Solutions need to be identified to sustain sensitive, acceptable and affordable TBE surveillance in all districts of Poland. Also, higher attention should be given to the diagnosis of TBE in the elderly.

## Background

Tick-borne encephalitis (TBE) is caused by TBE virus (TBEV). The virus is transmitted to susceptible individuals through tick bites or consumption of unpasteurized milk from recently infected animals [1]. TBE is restricted to geographical areas referred to as endemic foci [1,2]. Factors influencing survival of ticks, large mammals and transmission-competent rodents may explain the distribution of these foci and their changes over time [3,4]. Symptoms of TBE do not differ from other infections of the central nervous system (CNS). Thus laboratory confirmation of each suspected case is necessary. Due to their immature CNS, young children experience less severe symptoms than adults [1]. Compared with other age groups, the elderly present atypical symptoms and suffer from severe outcomes and higher case-fatality [1,5]. One third of infected adults develop flu-like symptoms. Of these, one third progresses to meningo-encephalitis following a 7-day symptom-free interval [1,6]. Twenty-six to 46% of infected persons who develop meningo-encephalitis suffer from post-encephalitic sequelae [1]. No specific anti-viral therapy has been developed, but safe and effective vaccines are available [7]. Primary immunisation consisting of three doses followed by booster doses administered 3–5 years apart is sufficient to acquire immunity [7,8]. Documentation of endemic foci allows development of focused vaccination recommendations [9,10]. However, to locate endemic foci, physicians must be aware of the disease and refer patients with signs of meningo-encephalitis for testing.

Poland (2010 population: 38 million) is divided into 16 provinces and 379 districts. TBE surveillance was implemented in 1970. Physicians notify meningo-encephalitis cases, including confirmed TBE cases. Vaccination is recommended for persons occupationally exposed to forests in endemic districts (i.e. farmers, foresters, soldiers). However, employers or individuals have to cover the vaccine cost [9]. The location of endemic areas for those recommendations is ascertained based on TBE reported rates. However, in 2004–2008 availability of serological diagnosis in only 39% of the country’s hospitals suggested that TBE remained undiagnosed in many provinces [11]. Furthermore, seroprevalence surveys among humans and animals suggested that TBE endemic foci existed in districts where no human cases had been reported [12,13].

Between March 2009 and April 2010, we implemented a pilot project of enhanced surveillance for TBE in selected Polish provinces. We aimed at describing patterns of the physician’s diagnosis, including referral for serological testing. In this paper, we summarize 13 months of enhanced TBE surveillance and assess the degree to which extension of serological testing to districts where it was not available led to identification of new endemic foci in Poland.

## Methods

We appointed epidemiologists that were delegated from the local and provincial health departments as local and provincial coordinators. Their role was to set up enhanced surveillance in selected wards admitting patients with aseptic meningo-encephalitis. Before the initiation of the project, we organized half-day training conferences for all project participants. The conferences covered lectures on tick-borne encephalitis and active surveillance methods.

### Case definitions

We defined a suspect TBE case as a patient hospitalized in a participating ward during the study period whose admission ICD-10 code indicated aseptic meningo-encephalitis or whose specimen was sent to the National Reference Laboratory (NRL) for TBE confirmation. We defined a confirmed TBE case as a suspected case in which detection of antibodies in serum (IgM and IgG) and/or in the cerebrospinal fluid CSF (IgG) confirmed current TBEV infection.

### Population under surveillance

Due to logistical constraints, we selected 11 out of 16 Polish provinces, including some with and without known endemic foci. First, we defined the upper limit for the number of hospital wards that could be included considering the available budget. Second, we added provinces following approval of provincial chief medical officers, and included hospital wards identified by a prior survey as routinely notifying aseptic meningo-encephalitis cases during the previous 5 years [11]. In these hospitals, we offered physicians the opportunity to send serum and CSF specimens from suspected cases for free confirmatory testing in the NRL. The decision to send samples for testing was made by hospital physicians. Local coordinators screened hospital registers for suspect cases and reported them weekly to provincial coordinators. For each suspect case, local coordinators filled out routine surveillance forms that included demographic, clinical and epidemiological information assessing possible exposure to ticks at the site of residence and elsewhere. Provincial coordinators collected weekly lists of suspect cases, the information on the number of requested tests, and surveillance forms. They validated and forwarded them to the central coordination team.

### Laboratory procedures

Serum specimens were diluted 1:100 and tested for IgM and IgG anti-TBE antibodies. CSF specimens were diluted 1:10 and tested for IgG anti-TBE antibodies. We used an ELISA test (FSME/ESME ELISA IgG/IgM Testkit, Genzyme Diagnostics VIROTECH, Germany), according to the manufacturer’s instructions. Results were expressed in “Vienna Units” (VE) defined as the ratio of absorbance (OD) of test specimen to the mean OD values of cut-off control, multiplied by 10. A specimen was considered positive if VE >11, negative if <9 and equivocal if 9 ≥ VE ≤11. Equivocal results were not considered for confirming cases. Specimens shipped to the laboratory were tested once or twice per week. Due to cross-reactivity with other flaviviruses pathogenic to humans, TBE ELISA positive results should by confirmed by more specific methods (ie., heamagglutination or neutralization tests) [1,14]. This is not performed routinely in Poland due to high cost and lack of other neuroinvasive flaviviral infections detected in humans to date.

### Data analysis

Choropleth maps were used to display the geographical location of TBE endemic districts, taking the Eurostat NUTS-4 administrative districts [15] as the unit of ascertainment. We calculated reported rates per 100,000 inhabitants by dividing the number of cases assigned to the presumed district of exposure by district mid-year census estimates. To account for uneven population density and uneven case clustering within the districts, we smoothed the maps of endemic districts by weighting reported rates of each district to the rates in adjacent districts. Using these established reported rates, we classified districts as non-endemic (< 0.1 cases per 100,000 inhabitants), low endemic (> = 0.1 to < 1), moderately endemic (> = 1 to < 5) and highly endemic (> = 5). We assigned urban districts (i.e., boundaries of larger towns) with the same endemicity status as the surrounding rural districts.

To assess the performance of enhanced surveillance, we calculated the proportion of TBE suspect cases, proportion of suspect cases referred for testing and proportion of positives, stratified by age group and 2004–2008 endemicity status of the district of hospital location.

We compared the endemicity status of districts in 2009 (using the data from a routine surveillance system that included additional cases identified through our project) with their status in 2004–2008 (using data from the routine surveillance system alone). We defined new endemic districts as those classified as non-endemic in 2004–2008, that were classified as endemic in 2009. We used STATA ver. 10 for data management and statistical analysis [16].

### Human subjects protection

The study used existing surveillance data. The only deviation from routine surveillance procedures was facilitating serum and CSF specimen testing for TBE at the NRL and planned storage of leftover specimens in a biobank for further investigation of neuroinvasive pathogens. The local coordinators obtained informed consent from each patient tested. They returned test results to clinicians immediately. The Ethical Committee of the National Institute of Public Health – National Institute of Health approved the enhanced surveillance protocol.

## Results

We implemented enhanced surveillance in 146 hospital wards during a median period of 11.2 months (mean 11.5 months, range: 7.2 to 14.2). Of 166,099 patients admitted to these wards, 1,585 were suspect cases [mean age: 35.0 years, 841 of males (53%); Table 1]. Hospital wards included in enhanced surveillance represented 81% of wards hospitalizing meningo-encephalitis patients in the 11 provinces and 49% of all wards in Poland.

**Table 1:** Characteristics of patients (n = 1,585) included as suspect tick-borne encephalitis cases, Poland, March 2009-April 2010

**Characteristic**		**Number of patients**	**%**
Age (years)	0-9	136	8.6
	10-19	325	20.5
	20-29	305	19.2
	30-39	222	14.0
	40-49	184	11.6
	50-59	193	12.2
	60+	220	13.9
Sex	Male	841	53.1
	Female	744	46.9
Residence type	Rural	712	45.0
	Urban	869	55.0
Month of onset	January	57	3.6
	February	79	5.0
	March*	89	5.6
	April*	82	5.2
	May	66	4.2
	June	133	8.4
	July	309	19.5
	August	273	17.2
	September	212	13.4
	October	138	8.7
	November	85	5.4
	December	62	3.9

* Summed cases reported in March-April 2009 and March-April 2010.

Of 1,585 suspect cases reported, physicians referred 1,106 (70%) for testing, including 1,028 serum and 808 CSF specimens. TBEV infection was confirmed in 246 patients, 173 based only on serum antibody detection, 6 based only on CSF antibody detection, and 77 based on antibody detection in both serum and CSF.

**Figure 1:**
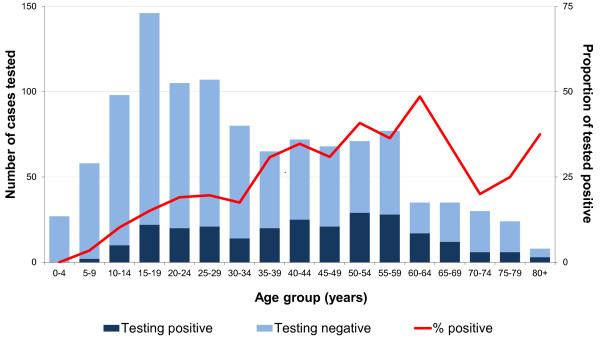
Number of suspected cases tested and percent positive by age group, Poland, March 2009-April 2010.

Physicians reported 12 suspect cases per 1,000 admitted patients <40 years old, of which 70% were tested (Table 2). For the same period, physicians reported 7 suspect cases per 1,000 admitted patients ≥40 years old, of which 70% were tested (Table 2). The proportion of patients that tested positive for TBE increased with age (Figure 1).

**Table 2:** Selected surveillance indicators measured in the observed population, by endemic status hospital region, 146 hospital units, March 2009-April 2010

**Characteristic**		**Admissions**	**Suspect TBE cases**					
			**Cases**	**Cases/1000 hosp.**	**Tested**	**%**	**Positive**	**% of those tested**
**Endemic status of hospital**	Non-endemic	89,309	639	7.2	378	59.2	17	4.5
	Low endemic	61,093	594	9.7	416	70.0	74	17.8
	Moderately endemic	8,731	135	15.5	117	86.7	32	27.4
	Highly endemic	6,966	217	31.2	195	89.9	133	68.2
**Age (years)**	0-9	34,851	136	3.9	89	65.4	3	3.4
	10-19	15,734	325	20.7	254	78.2	34	13.4
	20-29	12,866	305	23.7	209	68.5	39	18.7
	30-39	13,623	222	16.3	137	61.7	34	24.8
	40-49	15,633	184	11.8	143	77.7	49	34.3
	50-59	25,013	193	7.7	142	73.6	53	37.3
	60+	48,379	220	4.6	132	60.0	44	33.3
**Total**		166,099	1,585	9.5	1,106	69.8	256	23.2

*TBE* tick-borne encephalitis.

In hospitals located in districts classed as moderately and highly endemic in 2004–2008, physicians reported an average of 22 TBE suspected cases per 1000 hospitalized patients. Of those, 89% were tested for TBE. In hospitals located in districts classed as non-endemic and low endemic in 2004–2008, physicians reported 8 suspected cases per 1000 hospitalizations. Of those 64% were referred for testing (Table 2).

**Figure 2:**
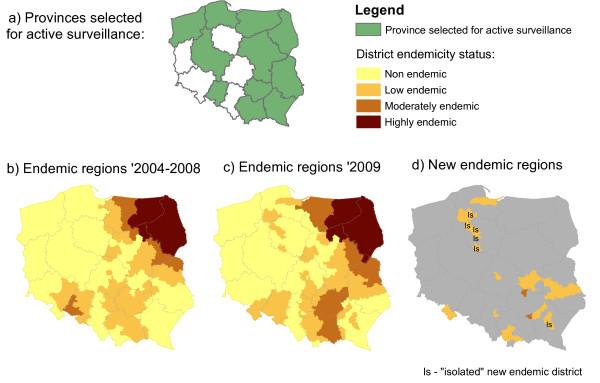
Geographical distribution of TBE endemic districts, Poland; a) Provinces selected for active surveillance; b) Endemic districts classified based on average reported rates during 2004–2008; c) Endemic districts classified based on reported rates recorded during 2009; d) Location of the newly identified endemic districts.

In 2009, 351 TBE cases were reported in Poland (0.92 per 100,000 inhabitants), 51% more than the annual median number of 233 cases (average annual reported rate 0.61) in 2004–2008. The status of 291 districts did not change between the two periods (Table 3). However, in 2009 we identified 38 new endemic districts. Of these, 31 were adjacent to known endemic foci in the Northeast and in the South and seven were isolated foci in the North and Centre of the country (Figure 2).

**Table 3:** Districts whose TBE endemicity status changed between 2004–2008 and 2009

**TBE endemic status of district**	**Number of districts in 2004-2008**	**Change of TBE endemic status in 2009**			
		**Non endemic**	**Low endemic**	**Moderately endemic**	**Highly endemic**
Non endemic	239	201 (84%)	36 (15%)	2 (1%)	-
Low endemic	95	14 (15%)	50 (53%)	31 (32%)	-
Moderately endemic	17	-	3 (18%)	12 (71%)	2 (11%)
Highly endemic	28	-	-	-	28 (100%)
All districts	379	215 (57%)	89 (23%)	45 (12%)	30 (8%)

*TBE* tick-borne encephalitis.

## Discussion

In 2009, we identified 38 new endemic districts. These may reflect old endemic districts that were undetected until 2009. Testing for TBE in patients with signs of meningo-encephalitis doubled in the entire country in 2009, compared with the annual number of specimens processed in previous years [11]. Moreover, according to the 2008 survey of hospitals admitting patients with meningo-encephalitis [11], districts newly identified as TBE endemic previously lacked routine diagnosis. One should not rule out that newly identified foci could also reflect the spread of TBE risk to new foci. Seven of the new endemic districts were situated away from previously known endemic foci, most notably in the Northwest of the country (Figure 2d). The possible existence of undetected TBE foci in these areas is supported by earlier seroepidemiological investigations [13]. New TBE foci can emerge, as reported in Sweden, Denmark and Norway [17-21]. Evidence from other European countries indicates that foci are extending particularly to the north [1,19,22] and higher altitudes [1,23]. These changes can be explained by complex interactions of environmental factors [3,4,18], changing patterns in climate and human behaviour, including travel, outdoor activities [18,22] and socio-economic changes [24,25]. For example, Godfrey and Randolph found a correlation between the 2009 high increases in TBE reported rates in Poland, Lithuania and Latvia and the high background levels of poverty [24].

Compared with endemic districts, referral for diagnosis was less common in districts that were non-endemic or low endemic in 2004–2008. According to the 2008 survey of hospitals admitting patients with meningo-encephalitis [11], physicians were reluctant to refer patients for TBE testing mostly due to the cost of serologic testing, considered as not justified by the available treatment options. However, our enhanced surveillance ensured availability of cost-free diagnosis in all participating wards. In addition, physicians in districts highly endemic in 2004–2008 referred patients with signs of meningo-encephalitis for testing 7-times more commonly than in districts considered non-endemic in 2004–2008. The decreased awareness of physicians in TBE-free districts may result in an underestimation of TBE outside known endemic areas. This might still leave possible endemic districts undetected.

Children and young adults were tested frequently, but few were positive for TBE. In contrast, older adults, including the elderly were rarely tested, yet a large proportion was positive. This may reflect the higher incidence of all cases of meningo-encephalitis diagnosis among children and adolescents. On the other hand, TBE leads to more severe disease in elderly patients, and is more difficult to diagnose because of atypical symptoms. This discrepancy between testing practices and age-specific risk may lead the Polish surveillance system to underestimate TBE reported rates.

This investigation had a number of limitations. First, 13-month enhanced surveillance may have been too short timeframe for reliable documentation of TBE foci. TBEV may circulate undetected for some time before the transmission to humans starts to occur, due to favourable weather conditions or socio-economic changes [24,26]. Therefore, the detection of sporadic cases could result in ascertainment of least populated districts as low endemic. To minimize this phenomenon, we smoothed maps of endemic districts, as was previously carried out routinely in Germany [27]. Second, we cannot rule out the possibility that the appearance of new foci reflected a real 2009 TBE peak in Poland. This is supported by a concomitant high TBE increase recorded in the Baltic countries that was triggered by an economic downturn [24]. In Poland it would be difficult to disentangle the real increase caused by in-work risk of poverty [24] with the effect of the unlimited testing offered to physicians through our enhanced surveillance in many regions where testing was not carried out. Third, coverage limited to 49% of hospital wards and lower awareness of physicians working in non-endemic districts may have led to geographical differences in surveillance performance. Thus, absence of evidence of disease in our project cannot be interpreted as a documentation of absence of TBE risk in several non-endemic districts, particularly in those where too few specimens were tested. Finally, cross-reactivity of serological tests used to confirm TBE [1,14] could theoretically have captured meningo-encephalitis cases caused by other flaviviruses. Thus, if other flavivirus were to be documented in Poland, laboratory diagnosis of TBE should be extended to neutralization testing.

## Conclusions

In conclusion, our enhanced surveillance enabled detection of new TBE endemic districts by offering systematic screening of all suspect TBE cases in a large proportion of Polish hospitals. However, lack of consistent testing in districts believed to be TBE-free remained an obstacle for mapping TBE risk. Although the disease is more severe in older adults and the elderly, more attention is given to the diagnosis of TBE in young patients. On the basis of these conclusions, we recommend (1) implementation of the national immunization policy in newly identified endemic districts; (2) uniform implementation of diagnostic procedures in all Polish hospitals and (3) more attention to diagnosis of TBE in the elderly.

## Appendix A – list of collaborators

Biała Podlaska: Marek Gałecki; Bielsk Podlaski: Ewa Zielenkiewicz-Madejska, Barbara Jarymowicz, Ewa Kierdelewicz; Biskupiec: Anna Giżewska; Busko-Zdrój: Grażyna Cieślik; Chełm: Maria Kornas- Rypina; Ciechanów: Dariusz Goryszewski, Italiana Pietrkiewicz; Dąbrowa Tarnowska: Zbigniew Martyka, Urszula Ostręga-Szeląg; Dębica: Urszula Gąsior; Dziekanów Leśny: Teresa Chmurska Motyka; Elbląg: Elżbieta Hajer, Jolanta Plewińska, Henryka Traczyk, Alicja Olszak-Rudzińska; Gdańsk: Anna Korczak-Rogoń, Aleksandra Goerick, Anetta Brzozowska, Maria Hlebowicz, Krystyna Malanowicz-Świerczyńska, Anna Lakomy; Giżycko: Anna Lachowicz-Wawrzyniak; Gniezno: Wojciech Jankowski; Hajnówka: Anna Nowicka-Ciełuszecka; Iława: Janusz Nowiński; Jarosław: Małgorzata Dankiewicz; Kalisz: Katarzyna Burchart-Adamczyk; Kielce: Wiesław Kryczka, Ewa Dutkiewicz; Konin: Ewa Dzielińska; Koszalin: Renata Batyra; Kościan: Dariusz Urbański; Kraków: Aleksander Garlicki, Jakub Loster, Barbara Lelek, Wojciech Kruk, Małgorzata Jawor-Bugajska, Barbara Postawa-Kłosińska, Małgorzata Pilawa; Kraśnik: Hanna Basista; Lubartów: Małgorzata Gierczak-Zdunek; Lublin: Krystyna Mitosek-Szewczyk, Iwona Halczuk, Tomasz Hasiec, Monika Gruszecka; Łańcut: Jerzy Sieklucki, Marcin Hawro; Łomża: Paweł Radom; Maków Podhalański: Dorota Kołodziejczyk; Miechów: Teresa Książek; Mielec: Józef Sznajder, Joanna Jankowska-Fietko; Nowy Sącz: Ewa Szczypuła; Nysa: Tadeusz Sarzyński; Olsztyn: Tomasz Siwek, Beata Zwiernik, Grzegorz Dałek, Krzysztof Nosek, Aniela Milicz-Płatek, Iwona Kibiłda, Małgorzata Czarniawska, Elżbieta Młynarczyk; Opole: Elżbieta Kluszczyńska, Krystyna Wierzbicka, Wiesława Błudzin, Ireneusz Bańburski, Gabriel Kryczka; Ostrołęka: Zbigniew Bieńkowski, Marcelina Bacharz, Grzegorz Kawałek, Piotr Paździor; Płock: Małgorzata Galon; Poznań: Lucyna Piechota, Beata Kramska, Dagmara Karolewska, Małgorzata Kędziora, Przemysław Augustyniak, Wojciech Służewski; Przemyśl: Danuta Malcher-Bober; Puławy: Elżbieta Burzmińska; Radom: Dorota Bielaczyc-Bęben, Andrzej Borysewicz; Radzyń Podlaski: Paweł Korzeniewski; Rzeszów: Magdalena Noga, Danuta Augustyn, Elżbieta Czyżyk, Barbara Baranowska, Anna Orłowska; Sandomierz: Piotr Sobolewski, Irena Łabudzka; Sanok: Stanisława Warzycha; Siedlce: Alicja Multan-Boguszewska, Grażyna Walęcik, Agata Włodek, Monika Czerska; Skarżysko-Kamienna: Mariola Grzegorek; Słupsk: Hanna Antonowicz, Marek Krysiak; Starachowice: Jadwiga Maciukajć, Dariusz Podwiak; Sucha Beskidzka: Jerzy Binek; Suwałki: Krzysztof Nowacki, Ewa Tynecka, Urszula Mojżesz; Szczecin: Jolanta Niścigorska-Olsen; Tarnów: Grażyna Zawada-Skrobisz, Lucyna Dziadoń; Wadowice: Wacław Słoboda; Wałcz: Mariusz Mróz; Warszawa: Małgorzata Zbroszczyk-Szczepaniak, Ewa Marcinkowska, Grażyna Cholewińska, Aleksandra Sosnowska-Dudek, Paweł Grąbczewski, Regina Podlasin, Dariusz Lipowski, Maria Olszyńska-Krowicka, Teresa Jackowska; Wejherowo: Wanda Czerska-Hladny.

## Competing interests

The authors declare that they have no competing interests.

## Authors’ contributions

PS conceived, designed and coordinated the study, analysed the data, and drafted the manuscript. AZH participated in the data analysis and helped to draft the manuscript. MH, RK, DL, LS, JS have made substantial contributions to the study coordination and acquisition of the data. HO participated in study design and coordination of the study, and helped to draft the manuscript. All authors read and approved of the final manuscript.

## Authors’ information

Members of the TBE enhanced surveillance working group are listed in Appendix A.

## References

[B1] Lindquist L Vapalahti O Tick-borne encephalitis Lancet 2008 371 1861 1871 10.1016/S0140-6736(08)60800-4 18514730

[B2] Donoso Mantke O Escadafal C Niedrig M Pfeffer M on behalf of the Working group for Tick-borne encephalitis virus Tick-borne encephalitis in Europe, 2007 to 2009 Euro Surveill 2011 16 19976 21968423 10.2807/ese.16.39.19976-en

[B3] Labuda M Randolph SE Survival strategy of tick-borne encephalitis virus: cellular basis and environmental determinants Zentralbl Bakteriol 1999 289 513 524 10.1016/S0934-8840(99)80005-X 10652718

[B4] Rizzoli A Hauffe HC Tagliapietra V Neteler M Rosà R Forest structure and roe deer abundance predict tick-borne encephalitis risk in Italy PLoS One 2009 4 e4336 10.1371/journal.pone.0004336 19183811 PMC2629566

[B5] Haglund M Gunther G Tick-borne encephalitis – pathogenesis, clinical course and long-term follow-up Vaccine 2003 21 Suppl 1 S11 S18 12628810 10.1016/s0264-410x(02)00811-3

[B6] Gustafson R Svenungsson B Forsgren M Gardulf A Granstrom M Two-year survey of the incidence of Lyme borreliosis and tick-borne encephalitis in a high-risk population in Sweden Eur J Clin Microbiol Infect Dis 1992 11 894 900 10.1007/BF01962369 1486884

[B7] WHO Publication Vaccines against tick-borne encephalitis: WHO position paper–recommendations Vaccine 2011 29 8769 8770 10.1016/j.vaccine.2011.07.024 21777636

[B8] Rendi-Wagner P Paulke-Korinek M Kundi M Wiedermann U Laaber B Kollaritsch H Antibody persistence following booster vaccination against tick-borne encephalitis: 3-year post-booster follow-up Vaccine 2007 25 5097 5101 10.1016/j.vaccine.2007.01.116 17555850

[B9] Stefanoff P Polkowska A Giambi C Levy-Bruhl D O’Flanagan D Dematte L Lopalco PL Mereckiene J Johansen K D’Ancona F VENICE project gatekeepers, contact persons group Reliable surveillance of tick-borne encephalitis in European countries is necessary to improve the quality of vaccine recommendations Vaccine 2011 29 1283 1288 10.1016/j.vaccine.2010.11.077 21145914

[B10] Braks M van der Giessen J Kretzschmar M van Pelt W Scholte EJ Reusken C Zeller H van Bortel W Sprong H Towards an integrated approach in surveillance of vector-borne diseases in Europe Parasit Vectors 2011 4 192 10.1186/1756-3305-4-192 21967706 PMC3199249

[B11] Stefanoff P Rogalska J Zajkowska J Czerska M Seroka W Czarkowski MP Surveillance of aseptic central nervous system infections in Poland: is it meeting its objectives? Euro Surveill 2011 16 19924 21801691

[B12] Cisak E Wójcik-Fatla A Zając V Sroka J Buczek A Dutkiewicz J Prevalence of tick-borne encephalitis virus (TBEV) in samples of raw milk taken randomly from cows, goats and sheep in eastern Poland Ann Agric Environ Med 2010 17 283 286 21186771

[B13] Stefanoff P Siennicka J Kaba J Nowicki M Ferenczi E Gut W Identification of new endemic tick-borne encephalitis foci in Poland - a pilot seroprevalence study in selected regions Int J Med Microbiol 2008 298 Suppl. 1 102 107

[B14] Holzmann H Diagnosis of tick-borne encephalitis Vaccine 2003 21 Suppl 1 S36 S40 12628812 10.1016/s0264-410x(02)00819-8

[B15] Regulation No 1059/2003 of the European Parliament and of the Council of 26 May 2003 on the establishment of a common classification of territorial units for statistics (NUTS) Official Journal of the European Union L 154, 21.6.2003, p. 1. Accessed on 12.11.2011 at http://eur-lex.europa.eu/LexUriServ/LexUriServ.do?uri=OJ:L:2003:154:0001:0041:EN:PDF

[B16] StataCorp Stata Statistical Software: Release 10 2007 College Station, TX: StataCorp LP

[B17] Haglund M Settergren B Heinz FX Gunther G The ISW-TBE Study Group Report of the meningitis program of the international scientific working group on TBE serological screening of patients with viral CNS infection of unknown etiology in the search of undiagnosed TBE cases Vaccine 2003 21 Suppl 1 S66 S72 12628817 10.1016/s0264-410x(02)00816-2

[B18] Jaenson TG Hjertqvist M Bergström T Lundkvist A Why is tick-borne encephalitis increasing? A review of the key factors causing the increasing incidence of human TBE in Sweden Parasit Vectors 2012 5 184 10.1186/1756-3305-5-184 22937961 PMC3439267

[B19] Fomsgaard A Christiansen C Bodker R First identification of tick-borne encephalitis in Denmark outside of Bornholm, August 2009 Euro Surveill 2009 14 19325 19758543

[B20] Skarpaas T Ljøstad U Sundøy A First human cases of tickborne encephalitis, Norway Emerg Infect Dis 2004 10 2241 2243 10.3201/eid1012.040598 15663873 PMC3323397

[B21] Andreassen A Jore S Cuber P Dudman S Tengs T Isaksen K Hygen HO Viljugrein H Anestad G Ottesen P Vainio K Prevalence of tick borne encephalitis virus in tick nymphs in relation to climatic factors on the southern coast of Norway Parasit Vectors 2012 5 177 10.1186/1756-3305-5-177 22913287 PMC3497858

[B22] Petri E Gniel D Zent O Tick-borne encephalitis (TBE) trends in epidemiology and current future management Travel Med Infect Dis 2010 4 233 245 20970726 10.1016/j.tmaid.2010.08.001

[B23] Danielová V Kliegrová S Daniel M Benes C Influence of climate warming on tickborne encephalitis expansion to higher altitudes over the last decade (1997–2006) in the Highland Region (Czech Republic) Cent Eur J Public Health 2008 16 4 11 18459472 10.21101/cejph.a3460

[B24] Godfrey ER Randolph SE Economic downturn results in tick-borne disease upsurge Parasit Vectors 2011 4 35 10.1186/1756-3305-4-35 21406086 PMC3063212

[B25] Stefanoff P Rosinska M Samuels S White DJ Morse DL Randolph SE A national case control study identifies human socio-economic status and activities as risk factors for tick-borne encephalitis in Poland PLoS One 2012 7 e45511 10.1371/journal.pone.0045511 23029063 PMC3446880

[B26] Randolph SE Asokliene L Avsic-Zupanc T Bormane A Burri C Gern L Golovljova I Hubalek Z Knap N Kondrusik M Kupca A Pejcoch M Vasilenko V Zygutiene M Variable spikes in tick-borne encephalitis incidence in 2006 independent of variable tick abundance but related to weather Parasit Vectors 2008 9 44 19068106 10.1186/1756-3305-1-44 PMC2614985

[B27] Editorial team Updated Risk areas for tick borne encephalitis in Germany Euro Surveill 2009 14 19236

